# West Nile Virus (WNV) Replication Is Independent of Autophagy in Mammalian Cells

**DOI:** 10.1371/journal.pone.0045800

**Published:** 2012-09-21

**Authors:** Rianna Vandergaast, Brenda L. Fredericksen

**Affiliations:** 1 Department of Cell Biology and Molecular Genetics University of Maryland, College Park, Maryland, United States of America; 2 Maryland Pathogen Research Institute, University of Maryland, College Park, Maryland, United States of America; Washington University, United States of America

## Abstract

Autophagy is a homeostatic process responsible for recycling cytosolic proteins and organelles. Moreover, this pathway contributes to the cell’s intrinsic innate defenses. While many viruses have evolved mechanisms to antagonize the antiviral effects of the autophagy pathway, others subvert autophagy to facilitate replication. Here, we have investigated the role of autophagy in West Nile virus (WNV) replication. Experiments in cell lines derived from a variety of sources, including the kidney, liver, skin, and brain, indicated that WNV replication does not upregulate the autophagy pathway. Furthermore, WNV infection did not inhibit rapamycin-induced autophagy, suggesting that WNV does not disrupt the authophagy signaling cascade. Perturbation of the autophagy pathway by depletion of the major autophagy factors Atg5 or Atg7 had no effect on WNV infectious particle production, indicating that WNV does not require a functional autophagy pathway for replication. Taken together, the results of our study provide evidence that WNV, unlike several other viruses of the family *Flaviviridae*, does not significantly interact with the conventional autophagy pathway in mammalian cells.

## Introduction

Viruses of the family *Flaviviridae* are small, enveloped, single-stranded, positive-sense RNA viruses. Members of this family include highly prevalent and medically important arthropod- or blood-borne viruses such as dengue virus (DENV), hepatitis C virus (HCV), Japanese encephalitis virus (JEV) and West Nile virus (WNV). Prior to the 1990s, WNV was not considered a major public health threat because infections were typically asymptomatic or resulted in a mild febrile illness. However, recent outbreaks within the Americas have been associated with a sharp increase in the incidence of severe neurological diseases, including meningitis, encephalitis, and acute flaccid paralysis (reviewed in [1], [2]). Treatment for severe cases of WNV is currently limited to supportive care, as specific therapies are not available. A more complete understanding of the factors that influence WNV replication and pathogenesis is therefore necessary to aid in the development of vaccines and antiviral therapies specific for WNV.

Recent evidence suggests that host autophagy pathways influence the replication and dissemination of multiple viruses. Autophagy is a multi-step process responsible for the bulk degradation and recycling of cytoplasmic components (reviewed in [3], [4]). The process begins with the formation of an isolation membrane, referred to as a phagophore, which sequesters cytoplasmic material, organelles, or invading pathogens. Two conserved ubiquitin-like conjugation systems, Atg12 and microtubule-associated protein 1A/1B light chain 3 (LC3), are essential for elongation of the phagophore into a double-membrane vesicle called the autophagosome. Conjugation of Atg12 to Atg5 facilitates complex formation with Atg16L1. The resulting heterotrimer associates with the phagophore and, with the help of Atg7 and Atg3, mediates conjugation of phosphatidylethanolamine to cytsolic LC3-I to produce LC3-II. This lipidated LC3-II is incorporated into the autophagosome and participates in cargo selection. Ultimately, autophagosomes fuse with lysosomes to generate autolysosomes, in which lysosomal proteases degrade the sequestered cargo.

Autophagy has the capacity to be both pro- and antiviral. For viruses such as VSV [5], Sendai virus [6], and HSV-1 [7], autophagy-mediated degradation of viral proteins helps limit viral replication and spread and promotes cell survival. In contrast, hepatitis B virus [8], poliovirus [9], coxsackievirus [10], [11], influenza virus [12], [13], chikungunya virus [14], and several members of the family *Flaviviridae,* including HCV [15]–[18], DENV [19]–[24], JEV [25], and Modoc virus [23], hijack components of the autophagy pathway to promote viral replication or dissemination. For viruses of the family *Flaviviridae*, evidence suggests that autophagy may promote replication through several mechanisms, which include limiting the innate antiviral response [26]–[28], promoting translation of the incoming viral genome [15], and providing additional energy as well as membrane structures for virus replication [17], [20], [21], [24], [25], [29]. Thus, manipulation of the autophagy pathway may be a conserved strategy used by members of the family to enhance replication.

In this study, we investigated the role of the autophagy pathway in WNV replication. We found that WNV infection did not upregulate the autophagy pathway. Exogenously-induced autophagy was also unaffected by WNV. Moreover, disruption of the autophagy pathway by depletion of Atg5 or Atg7, both of which are crucial for autophagosome development, had no effect on WNV replication. Together, our findings suggest that WNV, in contrast to HCV, DENV and JEV, does not interact with the autophagy pathway in mammalian cells. Hence, therapies that restrict autophagy are unlikely to be efficacious against all members of the *Flavivirus* family.

## Results

### Basal Levels of LC3B-II Vary between Cell Lines

**Figure 1:**
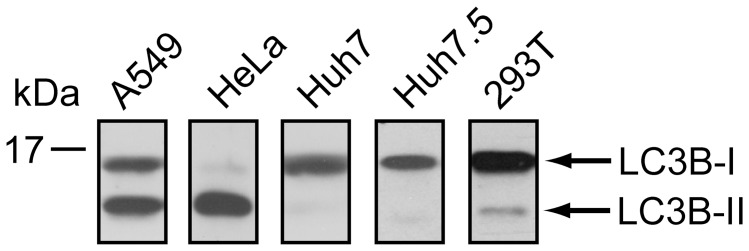
LC3B levels in various human cell lines. Whole cell lysates prepared from A549, HeLa, Huh7, Huh7.5, or 293T cells were subjected to immunoblot analysis by using anti-LC3B. Protein molecular size standards (in kilodaltons) are indicated on the left.

Several members of the family *Flaviviridae* subvert host autophagy pathways to enhance viral replication [15]–[25]. We hypothesized that WNV may also usurp the autophagy pathway to achieve optimal replication. Basal levels of autophagy vary dramatically between cell lines as well as culture conditions (reviewed in [30]). Therefore, we evaluated the basal level of autophagy in a variety of established cell lines in order to identify an appropriate cell line in which to assess WNV’s interactions with the autophagy pathway. Accumulation of lipidated LC3B-II, which serves as a marker for the formation of autophagosomes, was used to examine the autophagy pathway in A549, HeLa, Huh7, Huh7.5, and 293T cells grown under standard tissue culture conditions. High levels of LC3B-II were detected in both A549 and HeLa cells (Fig. 1, lanes 1 and 2), indicating that these cell lines have a high number of autophagosomes. In contrast, little to no LC3B-II was detected in Huh7, Huh7.5, and 293T cells (Fig. 1, lanes 3–5). Thus, of the cell lines examined, Huh7, Huh7.5, and 293T cells provided the most potential for detecting WNV-mediated upregulation of LC3B-II levels, and hence autophagy, under our tissue culture conditions.

### WNV does not Modulate the Autophagy Pathway in Established Cell Lines

**Figure 2:**
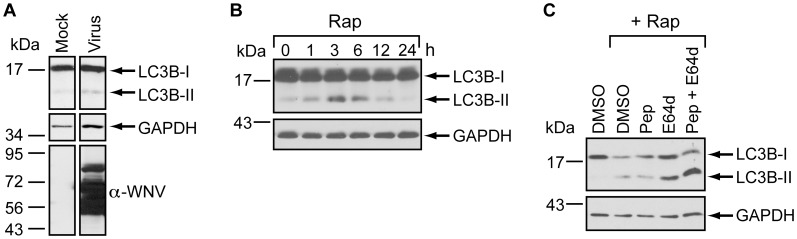
LC3B-II accumulation in 293T cells. (A) Effect of WNV infection on LC3-II levels. Whole cell lysates were prepared from mock-infected or WNV-infected (MOI = 3) 293T cells at 24 hours post-infection. Immunoblot anyalysis was used to determine steady-state levels of LC3B (top), GAPDH (middle), and WNV (bottom). (B) Effect of rapamycin on LC3B. Whole cell lysates prepared at the indicated times (h) from 293T cells treated with 100 nM rapamycin (rap) were subjected to immunoblot analysis to determine the steady-state levels of LC3B (top) and GAPDH (bottom). (C) Effect of protease inhibitors on LC3B-II levels. Whole cell lysates were prepared from 293T monolayers treated with rapamycin (Rap) in the presence or absence of pepstatin A (Pep) and/or E64d at 24 hours post-treatment. Lysates were subjected to immunoblot analysis as described in Panel B.

**Figure 3:**
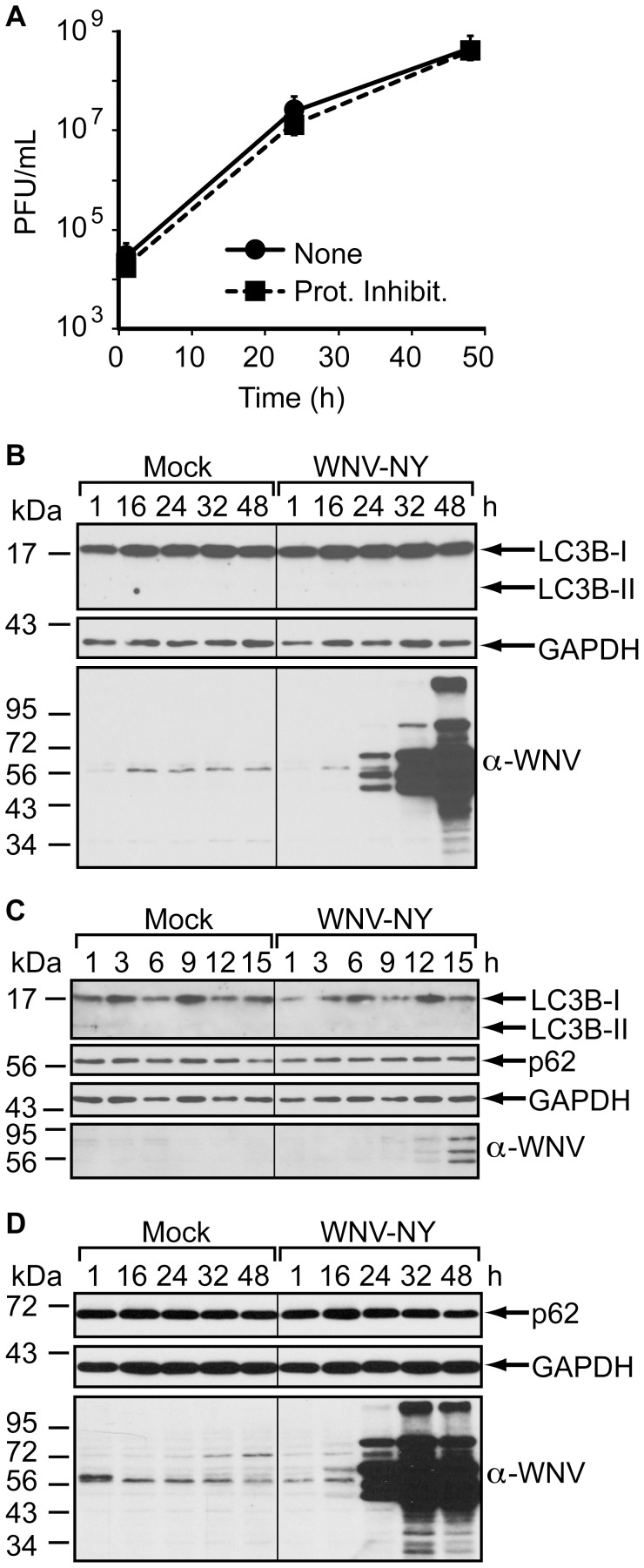
WNV-NY replication does not induce autophagy in 293T cells. (A) Effect of protease inhibitors on WNV replication. 293T cells infected with WNV-NY (MOI = 3) were incubated in the presence (Prot. Inhibit.) or absence (None) of 10 µg/mL Pepstatin A and E64d. Media was removed from cultures at the indicated times, cleared of cell debris, and titers determined by plaque assay on Vero cells. Values represent the average (± standard error) number of plaque-forming units (pfu) per mL from two independent experiments. (B) Effect of WNV-NY infection on steady-state levels of LC3B-II. 293T monolayers were mock-infected or infected with WNV-NY (MOI = 3). After infection, the inoculum was replaced with medium containing 10 µg/mL Pepstatin A and E64d. Whole cell lysates prepared at the indicated times (h) post-infection were subjected to immunoblot analysis for expression of LC3B (top), GAPDH (middle), and WNV (bottom). (C) Effect of WNV-NY infection on steady-state levels of LC3B-II and p62. 293T monolayers were mock-infected or infected with WNV-NY (MOI = 3) in the absence of protease inhibitors. Whole cell lysates prepared at the indicated times (h) post-infection were subjected to immunoblot analysis for expression of LC3B (top panel), p62 (second panel), GAPDH (third panel), and WNV (bottom panel). (D) Effect of WNV infection of levels of p62. 293T cells were mock-infected or infected with WNV-NY (MOI = 3) in the absence of protease inhibitors. Whole cell lysates prepared at the indicated times (h) post-infection were subjected to immunoblot analysis for expression of p62 (top), GAPDH (middle) or WNV (bottom).

To determine whether WNV induces autophagy, we monitored LC3B-II levels in 293T cells infected with WNV New York (WNV-NY), a highly pathogenic strain of WNV [31]. LC3B-II did not accumulate in WNV-NY infected cells at 24 hours post-infection (Fig. 2A), suggesting that autophagy was not upregulated. To verify that the autophagy pathway was intact in this cell line, we treated 293T cells with the autophagy-inducing drug rapamycin. LC3B-II levels increased within 3 hours of rapamycin treatment (Fig. 2B), confirming the capacity of these cells to respond to a known autophagic stimulus. However, by 6 hours post-treatment, LC3B-II levels began to decrease and only modest levels were present after 24 hours. This reduction in LC3B-II levels at later times after rapamycin treatment is consistent with previous reports that LC3B-II is degraded by autolysosomes in 293 cells undergoing starvation-induced autophagy [32]. Therefore, we measured LC3B-II levels in rapamycin-treated 293T cells grown in the presence or absence of the protease inhibitors Pepstatin A and/or E64d, which prevent degradation of LC3B-II [32]. LC3B-II accumulated in cells treated with both Pepstatin A and E64d (Fig. 2C), confirming that protease inhibitors are necessary to block LC3B-II turnover and to detect LC3B-II accumulation within this cell line. Thus, we monitored LC3B-II levels in WNV-NY-infected 293T cells in the presence of Pepstatin A and E64d. Importantly, the protease inhibitors did not alter either the kinetics of infectious particle production or the level of peak virus titers (Fig. 3A). Yet, even under conditions that block autophagic flux, WNV-NY did not cause LC3B-II accumulation (Fig. 3B). Furthermore, LC3B-II was not detected in WNV-infected cells at early times post-infection in the presence (data not shown) or absence of protease inhibitors (Fig. 3C). Thus, the lack of LC3B-II accumulation during WNV infection suggested that autophagy was not upregulated. To confirm these results, we also assessed steady-state levels of p62/SQSTM1, a cytoplasmic protein that is targeted to and degraded by autolysosomes (reviewed in [33]). Levels of p62/SQSTM1 were unaffected by WNV-NY infection at both early (Fig. 3C) and late times post-infection (Fig. 3D).

**Figure 4:**
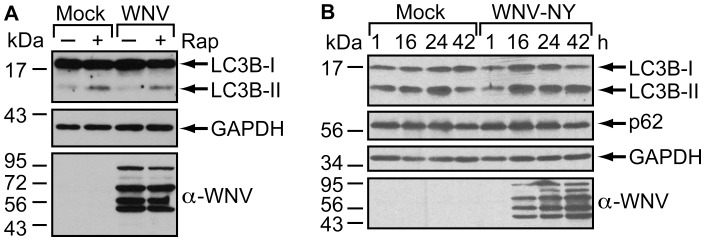
WNV does not interfere with autophagy induction. (A) Effect of WNV infection on rapamycin-induced LC3B-II accumulation. Mock- and WNV-NY- (MOI = 3) infected 293T monolayers were treated with rapamycin (rap) at 16 hours post-infection. Whole cell lysates were prepared 8 hours post-treatment with rapamycin and subjected to immunoblot analysis for expression of LC3B (top), GAPDH (middle), and WNV (bottom). (B) Effect of WNV infection on steady-state levels of LC3B-II in A549 cells. A549 monolayers were mock-infected or infected with WNV-NY (MOI = 3) in the absence of protease inhibitors. Whole cell lysates prepared at the indicated times (h) post-infection were subjected to immunoblot analysis for expression of LC3B (top panel), p62 (second panel), GAPDH (third panel), and WNV (bottom panel).

The inability to detect either LC3B-II accumulation or p62 depletion suggested that WNV-NY does not induce autolysosome formation in 293T cells. To rule out the possibility that WNV-NY actively impedes the autophagy pathway, we measured LC3B-II levels in WNV-NY-infected cells in the presence or absence of rapamycin. Rapamycin-induced accumulation of LC3B-II was similar in mock- and WNV-NY-infected cells (Fig. 4A), suggesting that WNV-NY does not inhibit exogenous stimulation of the autophagy pathway in 293T cells. Moreover, WNV infection did not reduce the high basal levels of LC3B-II in A549 cells (Fig. 4B), further suggesting that WNV does not block autophagosome formation.

**Figure 5:**
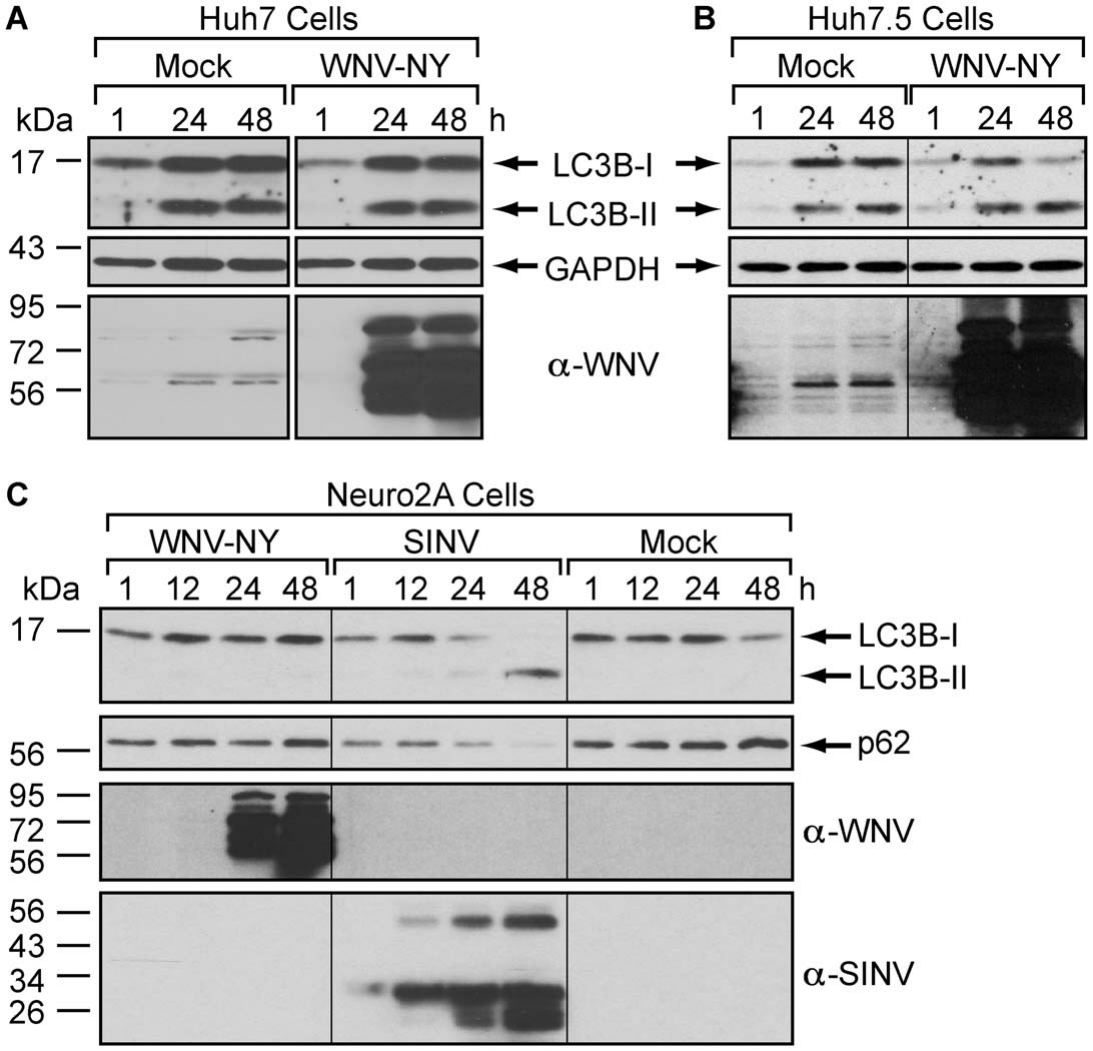
The autophagy pathway is not upregulated by WNV in established cell lines. (A and B) Effect of WNV infection on steady-state LC3B-II levels in Huh7 (A) or Huh7.5 (B) cells. Following mock or WNV-NY (MOI = 3) infection, cells were grown in medium containing 10 µg/mL Pepstatin A and E64d. Whole cell lysates prepared at the indicated times (h) post-infection were subjected to immunoblot analysis for expression of LC3B (top), GAPDH (middle), and WNV (bottom). (C) Effect of WNV and SINV infection of steady-state LC3B-II and p62 levels in Neuro2A cells. Neuro2A cells were mock-infected or infected with WNV (MOI = 3) or SINV (MOI = 5) in the absence of protease inhibitors. Whole cell lysates prepared at the indicated times (h) post-infection were subjected to immunoblot analysis for expression of LC3B (top panel), p62 (second panel), WNV (third panel), and SINV (bottom panel).

To confirm that the lack of a WNV-mediated induction of autophagy was not cell type specific, we examined the effect of WNV-NY on the autophagy pathway in several other established cell lines (Figure 5). Huh7 and Huh7.5 cell lines were selected, as these cell lines have been use to characterize the role of the autophagy pathway in the replication of other members of the family *Flaviviridae* [15]–[20], [22], [27], [29]. While increased LC3B-II levels were detected in WNV-NY-infected Huh7 and Huh7.5 cells at 24 and 48 hours post-infection in the presence of protease inhibitors, a concurrent increase in LC3B-II levels was detected in mock-infected controls (Fig. 5A and B). Thus, the accumulation of LC3B-II was independent of WNV-NY replication. Instead, the accumulation of LC3B-II was likely due to inhibition of LC3B-II turnover by the protease inhibitors, suggesting that these cell lines also maintain a high basal level of autophagy.

We also assessed the activity of the autophagy pathway in the mouse neuroblastoma cell line Neuro2A, which has been used to evaluate the effect of autophagy on the replication of another neurotropic virus, Sindbis virus [34]. Consistent with our previous findings, WNV-NY had no effect on the steady-state levels of LC3B-II or p62 in Neuro2A cells (Fig. 5C). In contrast, significant depletion of p62 and accumulation of LC3B-II was detected in Neuro2A cells infected with Sindbis virus (Fig. 5C).

**Figure 6:**
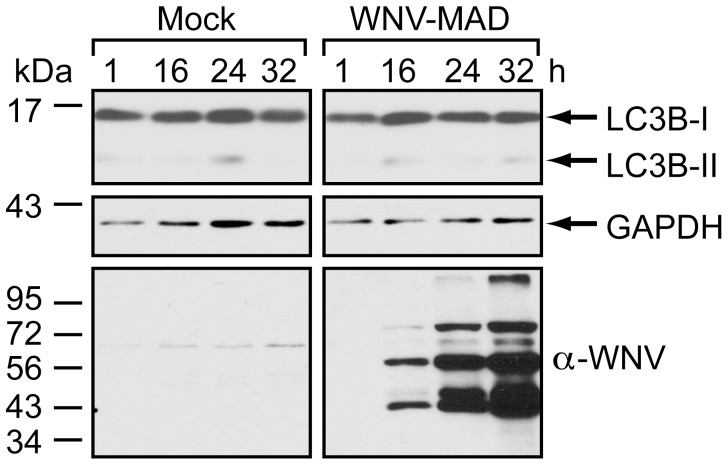
WNV-MAD78 infection does not induce autophagy. 293T monolayers were mock-infected or infected with WNV-MAD78 (MOI = 3). Following infection, cells were grown in medium containing 10 µg/mL Pepstatin A and E64d. Whole cell lysates prepared at the indicated times (h) post-infection were subjected to immunoblot analysis for LC3B (top), GAPDH (middle) and WNV (bottom).

Together, our findings suggested that replication of a virulent strain of WNV does not induce or enhance autophagosome formation. However, strain dependent differences in interactions with the autophagy pathway have been reported for both dengue virus and JEV [21], [24], [25]. In the case of JEV, a virulent strain was reported to induce lower levels of autophagy compared to an attenuated strain [25]. To determine whether WNV exhibits similar virulence-dependent variability in the induction of autophagy, we monitored autophagy following infection with a low pathogenicity strain of WNV, WNV-Madagascar (WNV-MAD78) [35], [36]. WNV-MAD78 infection did not induce LC3B-II accumulation (Fig. 6) or p62 degradation (data not shown) in 293T cells. Thus, neither pathogenic nor nonpathogenic strains of WNV upregulate the autophagy pathway.

### Primary Cell Lines do not Upregulate Autophagy in Response to WNV-NY Infection

**Figure 7:**
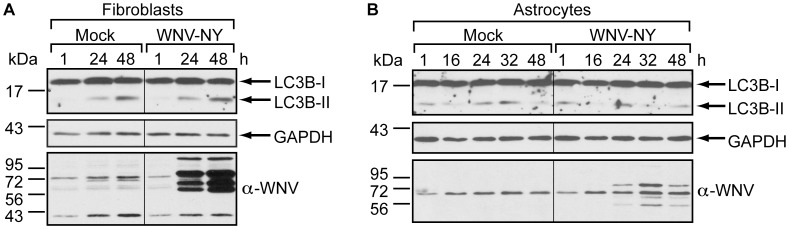
Primary human cell lines do not upregulate autophagy in response to WNV infection. (A-B) Effect of WNV infection on steady-state LC3B-II levels. Human foreskin fibroblasts (A) or human brain cortical astrocytes (B) were mock-infected or infected with WNV-NY (MOI = 3). Following infection, cells were grown in medium containing 10 µg/mL Pepstatin A and E64d. Whole cell lysates prepared at the indicated times (h) post-infection were subjected to immunoblot analysis for expression of LC3B (top), GAPDH (middle) and WNV (bottom).

To ensure that our findings were not biased by the use of immortalized cell lines, we also assessed WNV-NY’s ability to stimulate autophagy in two primary human cell lines, foreskin fibroblasts (HFF) and brain cortical astrocytes (HBCAs) (Fig. 7). Skin fibroblasts are one of the first cell types encountered by WNV following inoculation by the mosquito vector and therefore represent the first barrier to WNV dissemination during the natural course of infection. A modest increase in LC3B-II levels was observed in WNV-NY-infected HFFs at 24 and 48 hours after infection (Fig. 7A). However, as we observed in Huh7 and Huh7.5 cells, a comparable increase was also detected in mock-infected cells, indicative of WNV-independent upregulation of autophagy. Similar results were observed when cells were infected with insect cell-derived WNV-NY grown in mosquito C6/36 cells (data not shown), indicating that the lack of stimulation was independent of the source of the virus.

Activation of the autophagy pathway by Sindbis virus is cytoprotective and essential for neuronal cell survival [34]. Although WNV infection of neuronal cells is cytopathic both *in vitro* and *in vivo*, minimal cytopathic effect (CPE) is detected during productive WNV infection of primary human and mouse astrocytes (unpublished data Husmann and Fredericksen and [37], [38]). To determine whether the lack of CPE in astrocytes is due to autophagy-mediated cytoprotective effects, we monitored LC3B-II levels in WNV-NY-infected primary human brain cortical astrocytes (HBCAs). As with the other cell lines tested, comparable levels of LC3B-II were detected in mock- and WNV-NY-infected HBCAs throughout the course of infection (Figure 7B). Thus, the lack of WNV-induced CPE in astrocytes is not likely due to autophagy-mediated cytoprotection.

### Disruption of the Autophagy Pathway does not Alter WNV Replication

**Figure 8:**
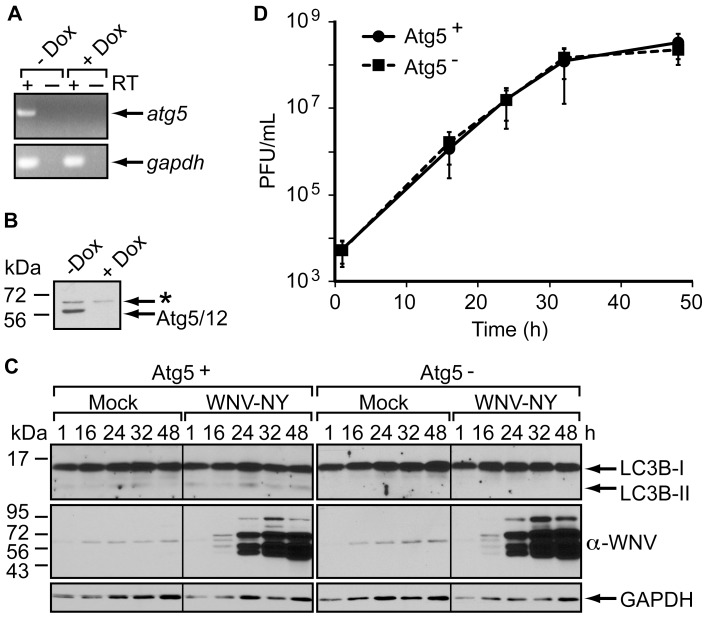
Atg5 is not required for WNV replication. (A) Atg5 mRNA in m5-7 cells. Standard RT-PCR (+ RT) was used to detect the presence of *atg5* (top) or *gapdh* (bottom) mRNA in total RNA extracted from m5-7 cells grown in the presence or absence of 10 ng/mL doxycycline (Dox). PCR products were analyzed by agarose-gel electrophoresis. (B) Atg5 protein expression in m5-7 cells. Whole cell lysates prepared from m5-7 cells grown in the presence or absence of doxycycline were subjected to immunoblot analysis for Atg5 expression. A non-specific band (*) was also detected with this antiserum. (C) Immunoblot analysis of WNV-NY-infected m5-7 cells. m5-7 monolayers grown in the presence (Atg5^-^) or absence (Atg5^+^) of 10 ng/mL doxycycline were mock-infected or infected with WNV-NY (MOI = 3). After infection, inoculum was replaced with medium containing 10 µg/mL Peptastatin and E64d with or without 10 ng/mL doxycycline. Whole cell lysates were prepared at the indicated times (h) post-infection and subjected to immunoblot analysis for expression of LC3 (top), WNV (middle) and GAPDH (bottom). (D) WNV growth curves in the presence and absence of Atg5. Culture supernatants were recovered from the cells in Panel C at the indicated times (h) and the viral titers determined by plaque assay on Vero cells. Values represent the average (± standard error) number of plaque-forming units (pfu) per mL from four independent experiments.

To determine whether WNV replicates independently of the classical autophagy pathway, we compared WNV-NY replication in the presence and absence of a functional autophagy pathway. To inhibit autophagy, we used an immortalized Atg5−/− mouse embryonic fibroblast (MEF) cell line, called m5-7, that is stably transfected with a plasmid encoding the wild-type *atg5* gene under the control of a doxycycline-repressible promoter [39]. Repression of *atg5* expression by doxycycline (dox) was confirmed at both the RNA (Fig. 8A) and protein level (Fig. 8B). As previously reported [39], the monomeric form of Atg5 was difficult to detect in this cell line, but the Atg5-Atg12 complex was readily detected in the absence of doxycycline (Fig. 8B). As observed in the human cell lines, WNV-NY did not induce LC3B-II accumulation in m5-7 cells expressing Atg5 (dox-) (Fig. 8C). Moreover, WNV-NY replicated to similar levels in the presence (Atg5-) and absence (Atg5+) of doxycycline (Fig. 8D), indicating that autophagy does not influence WNV replication in immortalized MEFs.

**Figure 9:**
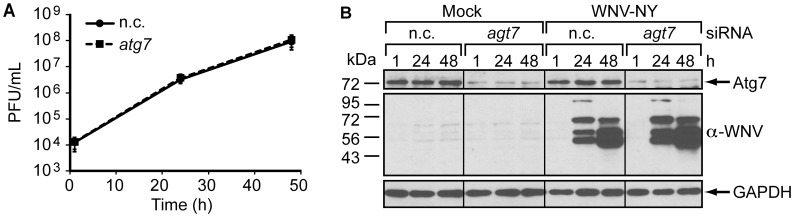
Autophagy does not influence WNV replication in 293T cells. (A) Infectious particle production in 293T cells following RNAi against *atg7*. 293T monolayers transfected with non-coding (n.c.) siRNA or siRNA targeting *atg7* were mock-infected or infected with WNV-NY (MOI = 3). Culture supernatants were recovered at the indicated times (h) and the viral titers determined by plaque assay on Vero cells. Values represent the average (± standard deviation) number of plaque-forming units (pfu) per mL from three independent experiments. (B) Immunoblot analysis of 293T cells following RNAi against *atg7*. Whole cell lysates prepared from the cells in Panel A were subjected to immunoblot analysis for expression of Atg7 (top), WNV (middle) and GAPDH (bottom).

To verify that WNV replication is unaltered by autophagy in human cells, we monitored WNV replication in 293T cells transfected with siRNA targeting a second component of the autophagy conjugation system required for membrane expansion, Atg7 (reviewed in [3]). WNV-NY growth curves were similar in cells transfected with an siRNA pool targeting *atg7* and control cells transfected with a non-coding siRNA pool (Fig. 9A). Immunoblot analyses confirmed that Atg7 expression was substantially reduced in cells transfected with the *atg7*-targeting siRNA pool (Fig. 9B). Thus, WNV replicates independently of a functional autophagy pathway.

## Discussion

Constitutive basal levels of autophagy play an important role in cellular homeostasis by removing protein aggregates and damaged organelles. However, autophagy is also upregulated in response to a variety of stresses, including infection with many different viruses. While starvation induces a nonspecific upreglation of protein degradation, pathogens stimulate a highly selective form of autophagy referred to as xenophagy (reviewed in [40], [41]). The xenophagy pathway interacts with components of the innate intracellular antiviral program to specifically target viral proteins or particles for degradation. Degradation of viral components by this specialized autophagy pathway can result in lower viral levels or may promote cell survival by preventing the accumulation of viral proteins that are toxic to the cell (reviewed in [40], [41]). For several viruses, disruption of the autophagy pathway can lead to increased viral loads.

Not surprisingly, many viruses have evolved mechanisms to either evade or usurp the autophagy pathway. Recent reports suggest that autophagy plays an integral role in the replication of several members of the family *Flaviviridae*. DENV, HCV, JEV, and Modoc virus upregulate autophagy in a variety of cell lines [15]–[18], [20]–[26], [42]. Moreover, autophagy induction enhances the replication of these viruses and disruption of autophagy results in decreased progeny virus production [16]–[21], [23]–[28]. Evidence suggests that autophagy aids in the replication of these viruses through multiple mechanisms. During HCV infection, autophagy impairs the innate antiviral response [26]–[28] and may promote translation of the incoming viral genome [15]. Alternatively, autophagy induced by DENV-2 increases lipid droplet processing, effectively increasing β-oxidation and energy available for replication [20]. Co-localization of HCV and DENV proteins with autophagic markers suggests that autophagic vesicles may also serve as sites of viral genome replication [17], [21], [24], [29].

Due to the role of autophagy in promoting HCV, DENV, and JEV replication, we hypothesized that the autophagy pathway may also promote WNV replication. However, our study indicated that neither a pathogenic nor nonpathogenic strain of WNV upreglulates autophagy. Our findings have also demonstrated that WNV infection does not impede autophagosome formation, as virus infection did not inhibit exogenous stimulation of autophagy by rapamycin or virus-independent LC3-II accumulation in Huh7, Huh7.5, A549, and HFF cells. Moreover, the lack of accumulation of LC3B-II in WNV-infected 293T cells in the absence of protease inhibitors (Fig. 2A and data not shown) indicated that WNV does not block autophagosome maturation and clearance. Thus, WNV infection does not alter autophagic flux in mammalian cells. We also demonstrated that inhibition of autophagy through depletion of the essential autophagy factors Atg5 or Atg7 had no effect on WNV replication. Together, our data suggested that, in contrast to other members of the family *Flaviviridae*, WNV does not utilize the autophagy pathway to enhance replication. Differences in the capacity of viruses within the same family to interact with autophagy have also been observed for members of the picornavirus family. Both poliovirus and coxsackievirus utilize the autophagy pathway to enhance replication, while human rhinovirus type2 does not [9]–[11], [43]. Therefore, our findings provide further evidence that viral interactions with the autophagy pathway are not conserved within families.

Based on our study, we cannot rule out the possibility that autophagy plays a role in WNV replication or dissemination *in vivo* in the mammalian or avian host or the mosquito vector. Because insects lack an adaptive immune response, autophagy may play a more prominent role in controlling viral infections in the mosquito. Indeed, the autophagy pathway is essential for controlling VSV infection in *Drosophila* [5]. Our data also does not exclude the possibility that WNV utilizes specific components of the autophagy pathway, other than Atg5 or Atg7, for viral replication. Nevertheless, our study demonstrated that WNV does not interact with the conventional autophagy pathway in mammalian cells, suggesting that inhibition of autophagy is not an effective strategy for controlling WNV infections in humans.

## Materials and Methods

### Cell Lines

Vero (kindly provided by Michael Gale Jr., University of Washington, Seattle, WA), 293T (GeneHunter, Nashville, TN), A549 (Sigma, St. Louis, MO), HeLa (kindly provided by Julie Pfeiffer, University of Texas Southwestern Medical Center, Dallas TX), Huh7 (kindly provided by Michael Gale Jr., University of Washington, Seattle, WA), Huh7.5 (Apath, Brooklyn, NY), human foreskin fibroblast (a kind gift from Dr. Alison McBride, National Institutes of Health, Bethesda, MD) [44], human brain cortical astrocyte (Cell Systems, Kirkland, WA ), and Neuro2A [45] cell lines were maintained at 37°C and 5% CO_2_ in Dulbecco’s modified Eagle medium (DMEM) (Mediatech) supplemented with 10% fetal bovine serum (FBS) (BioWhittaker), nonessential amino acids, 4.5 mg/L glucose, L-glutamine, and sodium pyruvate, 10 U/mL penicillin, 10 µg/mL streptomycin, and 25 ng/mL amphotericin B (complete DMEM). The mouse embryonic fibroblast (MEF) cell line m5-7 (a kind gift from Noboru Mizushima) [39], [46], in which Atg5 expression is repressed by tetracycline or doxycycline, was maintained in complete DMEM with or without 10 ng/mL doxycycline hyclate (MP Biomedicals).

### Viruses

Working stocks of WNV-NY strain 3356 were generated from the infectious clone pFLWNV [31]. Briefly, infectious particles were recovered as previously described [31], passaged once in 293 cells at a low multiplicity of infection (MOI), and subsequently passaged in Vero cells. WNV-MAD78 was obtained from the World Reference Center of Emerging Viruses and Arboviruses. Lyophilized virus was resuspended in complete DMEM+20% FBS, amplified once in Vero cells and plaque purified. Viral stocks were amplified once in 293 cells at a low MOI and working stocks were generated by passaging once in Vero cells. All viral stocks were aliquoted and stored at –80°C. Titers of viral stocks were determined by plaque assay on Vero cells (see below). A working stock of Sindbis virus TE (SINV) [47] was a kind gift from Diane Griffin.

### Viral Infections

Cultures of the indicated cell lines were washed once with DMEM and infected with WNV or SINV a multiplicity of infection (MOI) of 3 (WNV) or 5 (SINV). After 1 hour at 37°C, the inoculum was replaced with complete DMEM and cells were incubated at 37°C. Culture supernatants were collected at the indicated time points.

### Plaque Assays

Culture medium from infected monolayers was collected and clarified through centrifugation at 1,500 x g. Monolayers of Vero cells in six well plates were washed one time with DMEM followed by the addition of serial dilutions of viral samples. The cells were incubated in a 5% CO_2_ incubator for 1 hour at 37°C with rocking, the inoculums removed, and a 0.9% agarose-complete DMEM overlay added. Cell monolayers were incubated for 48 hours and a second overlay of agarose-complete DMEM containing 0.003% neutral red (MP Biomedicals) was added. Plaques were counted 4 (WNV-NY) or 7 (WNV-MAD) days after initial inoculation.

### Small Molecules

The protease inhibitors E64d (Sigma) and Pepstatin A (Fisher Scientific) were dissolved in dimethyl sulfoxide (DMSO) and diluted to a final concentration of 10 µg/mL in complete DMEM. The autophagy-inducer rapamycin (VWR) was dissolved in DMSO and diluted to a final concentration of 100 nM in complete DMEM.

### Immunoblot Analysis

Cell monolayers were washed twice with modified DPBS (Hyclone), and lysed with Ripa buffer (10 mM Tris pH 7.4, 150 mM NaCl, 0.02% NaN_3_, 1% sodium deoxycholate, 1% Triton X-100, 0.1% sodium dodecyl sulfate [SDS], and 1X protease inhibitors [Sigma]). Proteins (10–20 µg) were subjected to SDS-polyacrylamide gel electrophoresis (PAGE) and transferred to nitrocellulose membranes. Membranes were incubated with the following antiserum: polyclonal rabbit anti-LC3B (1∶3000) (Abcam; ab51520), monoclonal mouse anti-p62/SQSTM1 (1∶1000) (Abnova; M01 clone 2C11), polyclonal rabbit anti-GAPDH (1∶5000) (Abcam; ab36845), polyclonal mouse anti-WNV (1∶1000) (Arbovirus Resource Center; T35570), mouse anti-SINV (1∶500) (ATCC), monoclonal mouse anti-ATG5 (1∶2000) (MBL International Corporation; M153), and polyclonal rabbit anti-ATG7 (1∶500) (ProSci Inc; 3617).

### Reverse Transcription-PCR

Total mRNA was extracted from m5-7 monolayers using TriZOL Reagent (Invitrogen) as per the manufacturer’s directions. Following treatment with TurboDNase (Applied Biosystems), mRNA was used as a template for reverse transcription with random hexamers (Applied Biosystems) and M-MLV (New England Biolabs). The resulting cDNA was used as template for PCR with the following primer sets: mouseGAPDH(s), 5′-AAG GTC GGT GTG AAC GGA TTT-3′ and mouseGADPH(as) 5′-ATT TGC CGT GAG TGG AGT CAT AC-3′ or Atg5(s), 5′-GAT GTG CTT CGA GAT GTG TGG-3′ and Atg5(as), 5′-GTT GGC TGG GGG ACA ATG-3′. PCR products were analyzed by agarose-gel electrophoresis followed by staining with ethidium bromide.

### siRNA Transfections

siRNA was introduced into cells using reverse transfection. Briefly, 25 pmol of a non-coding siRNA pool or an siRNA pool directed against *atg7* (Dharmacon, D-001810-10-05 and L-020112-00-0005) was diluted in 100 µL OptiMEM medium (Invitrogen), placed in wells of a 24-well plate, and mixed with 1 µL Lipofectamine 2000 (Invitrogen). 293T cells were resuspended at 1.2×10^5^ cells/mL in complete DMEM +10% FBS without antibiotic/antimycotic and 500 µL was added to each well. Cells were incubated at 37°C for 46 hours prior to being used for infection experiments.
